# Immune checkpoint status and exhaustion‐related phenotypes of CD8^+^ T cells from the tumor‐draining regional lymph nodes in breast cancer

**DOI:** 10.1002/cam4.6802

**Published:** 2023-12-08

**Authors:** Izel Yilmaz, Ece Tavukcuoglu, Utku Horzum, Kerim Bora Yilmaz, Melih Akinci, Mehmet Ali Gulcelik, Haluk Barbaros Oral, Gunes Esendagli

**Affiliations:** ^1^ Department of Medical Immunology, Institute of Health Sciences Bursa Uludag University Bursa Turkey; ^2^ Department of Basic Oncology Hacettepe University Cancer Institute Ankara Turkey; ^3^ Department of General Surgery, Gulhane Training and Research Hospital University of Health Sciences Ankara Turkey; ^4^ Department of Medical and Surgical Research Hacettepe University Institute of Health Sciences Ankara Turkey; ^5^ Department of Immunology, Faculty of Medicine Bursa Uludag University Bursa Turkey

**Keywords:** breast cancer, cancer biology, microenvironment, surgical oncology

## Abstract

**Background:**

Functional status of T cells determines the responsiveness of cancer patients to immunotherapeutic interventions. Even though T cell‐mediated immunity is inaugurated in the tumor‐adjacent lymph nodes, peripheral blood has been routinely sampled for testing the immunological assays. The purpose of this study is to determine the immune checkpoint molecule expression and the exhaustion‐related phenotype of cytotoxic T cells in the regional lymph nodes from breast cancer patients.

**Patients and methods:**

Multicolor immunophenotyping was used to determine the expression of PD‐1, TIM‐3, LAG3, CTLA‐4, CCR7, CD45RO, CD127, CD25, CXCR5, and ICOS molecules on CD3^+^CD4^−^CD56^−^CD8^+^ cytotoxic T cells freshly obtained from the lymph nodes and the peripheral blood samples of the breast cancer patients. The results were assessed together with the clinical data.

**Results:**

A population of cytotoxic T cells was noted with high PD‐1 and CXCR5 expression in the lymph nodes of the breast cancer patients. Co‐expression of PD‐1, CXCR5, TIM‐3, and ICOS indicated a follicular helper T cell (Tfh)‐like, exhaustion‐related immunophenotype in these cytotoxic T cells. Only a minor population with CTLA‐4 and LAG3 expression was noted. The PD‐1^+^CXCR5^+^ cytotoxic T cells largely displayed CD45RO^+^CCR7^+^ central memory markers. The amount of CXCR5‐expressing PD‐1^−^ cytotoxic T cells was elevated in the lymph nodes of the patients.

**Conclusion:**

The regional lymph nodes of breast cancer patients harbor Tfh‐like exhausted cytotoxic T lymphocytes with high PD‐1 and TIM‐3 checkpoint molecule expression. The immunological conditions in the regional lymph nodes should be implicated for immune checkpoint immunotherapy (ICI) of cancer.

## Graphical abstract

The regional lymph nodes neighboring the tumor tissue harbor a cytotoxic T lymphocyte subset with the immunophenotypic characters of Tfh‐like exhausted cells with high PD‐1 and TIM‐3 checkpoint molecule expression which may modulate the outcomes of immunotherapeutic interventions in breast cancer.

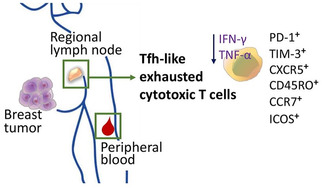

## 1 INTRODUCTION

CD8^+^ T cells have an essential role in the elimination of tumor cells.^1^, ^2^ Nevertheless, prolonged exposure to antigens and overexpression of the inhibitory receptors reduce the capacities of cytotoxicity, cytokine secretion, and proliferation; therefore, CD8^+^ T cells acquire a hyporesponsive state.^3^, ^4^, ^5^ The blockade of inhibitory receptors such as programmed death‐1 (PD‐1), T‐cell immunoglobulin mucin‐3 (TIM‐3), and cytotoxic T lymphocyte‐associated protein 4 (CTLA‐4) forms the basis of immune checkpoint immunotherapy (ICI) which aims to reactivate T cell‐mediated antitumor immunity.^4^, ^6^ However, the success of immunotherapy has been limited in breast cancer.^7^ Culminating evidence showed that not only the tumor‐infiltrating lymphocytes but also the T cells found in the lymphoid structures and in the tumor‐draining lymph nodes should be reinvigorated for achieving clinical response.^8^, ^9^ The lymph nodes serve as the sentinels for the initiation and expansion of the antigen‐specific T‐cell reactions^10^; however, the checkpoint status in these unique immune organs remains to be better elucidated in breast cancer.

Exhausted T cells (T_EX_) have been recognized with distinct epigenetic signatures and transcriptional profiles.^11^ Nevertheless, only a limited number of surface protein markers, which are not essentially specific for T‐cell subsets at distinct stages of exhaustion, are available for inferring T‐cell hyporesponsiveness.^12^, ^13^ For instance, the progenitor CD8^+^ T_EX_ cells have also been acknowledged with stem‐like,^14^, ^15^ memory‐like,^16^ and follicular helper T cell (Tfh)‐like features.^17^ The progenitor CD8^+^ T_EX_ cells can be identified with CXCR5, and PD‐1 markers shared by the CD4^+^ Tfh subset that frequently reside in the follicular structures of secondary lymphoid organs.^18^ The inducible costimulatory molecule (ICOS) and TIM‐3, which is another inhibitory receptor associated with the exhaustion, have also been associated with Tfh cells.^19^, ^20^ A few studies claimed the contribution of CXCR5^+^CD8^+^ Tfh‐like cells to germinal center reactions and antibody production, a function like that of CD4^+^ Tfh cells.^21^, ^22^ Elevated percentages of CXCR5^+^CD8^+^ T cells are reported in the circulation or in the tumor tissues of B cell lymphoma, hepatocellular carcinoma, colorectal cancer, and pancreatic cancer patients.^23^, ^24^, ^25^, ^26^, ^27^ In hepatocellular carcinoma, CXCR5^+^CD8^+^ T cells were frequently PD‐1‐positive and possessed higher proliferation and cytolytic capacities compared to those of CXCR5^−^CD8^+^ T cells.^23^

In cancer, the antitumor responses become dysregulated and inefficient in the lymph nodes which serve as a primary site for antigen presentation and T‐cell stimulation.^28^ Hence, this study aims to explore the immune checkpoint molecule status and exhaustion‐related facets of the CD8^+^ T cells found in the circulation and in the lymph nodes. Here, a subpopulation of CXCR5^+^PD‐1^+^ cytotoxic T cells was identified with high TIM‐3, ICOS, and CD45RO expression in the lymph nodes from breast cancer patients but showed limited association with the clinical features of the patients.

## 2 METHODS

### 2.1 Patients and healthy controls

The lymph node specimens removed as a part of breast cancer surgery were freshly obtained in physiological saline solution and delivered following an examination by a pathology specialist (*n* = 25). The lymph nodes sampled for this study were determined as nonmetastatic by conventional light microscopy. The absence of metastatic cancer cells was confirmed by reverse‐transcription polymerase chain reaction for epithelial cell adhesion molecule (EpCAM) and epithelial cadherin (E‐cadherin) genes (data not shown). Out of 25 lymph nodes, only two were PCR‐positive for these epithelial markers. Peripheral blood samples from the patients and the healthy volunteers (*n* = 11) were collected in the tubes containing 0.1% ethylenediaminetetraacetic acid tetrasodium (EDTA). Of total 25 patients, 5 received neoadjuvant chemotherapy prior to the surgery, and 6 were positive for human epidermal growth factor receptor 2 (HER‐2). A large portion of the patients (*n* = 22) were diagnosed with clinically early‐stage (0–II) cancer. Detailed information on patients is given in Table 1. The individuals with further medical complications or comorbidities and who were under medications other than cancer therapy were not enrolled in the study. This study was approved by the local ethical committee (Approval no: 2020/110) and a signed informed consent was obtained from the volunteers.

**TABLE 1:** Clinical properties of breast cancer patients and healthy controls.

	Healthy controls	Breast cancer patients
Total number (*n*)	11	28 (PB, 24; LN, 25)
Age median (min–max)	30 (24–50)	48 (28–79)
Neoadjuvant therapy		7
Naïve patient		21
Clinical stage		
0		3
I		10
II		12
III		3
Histopathological grade		
I		6
II		13
III		9
HER2‐positive		7
HER2‐negative		21
Lymph node status		
N0		12
N1(mi)		3
N1≤		10

Abbreviations: LN, lymph node; PB, peripheral blood.

### 2.2 Cell isolation and immunophenotyping

The lymph node specimens were mechanically dissociated and filtered through a 40 μm pore‐sized mesh. For peripheral blood samples, mononuclear cells were isolated after 1.077 g/mL density gradient separation (Ficoll™) (Sigma). The cells were washed and resuspended in RPMI‐1640 (Biological Industries) cell culture medium supplemented with 10% fetal bovine serum (FBS) (Biowest), 1% penicillin–streptomycin solution (Biological Industries). Then, the cells were counted and labeled with fluorochrome‐conjugated antihuman monoclonal antibodies (100 ng/10^6^ cells in 0.1 mL) reactive to CD4 (OKT4), CD8 (SK1), CD3 (SK7), CD56 (MEM‐188), CD45 (UCHL1), PD‐1 (EH12.2H7), CXCR5 (J25D4), CCR7 (G043H7), CD45RO (UCHL1), CTLA‐4 (L3D10), LAG3 (11C3C65), TIM‐3 (F38‐2E2), CD25 (M‐A251), CD127 (A019D5), and ICOS (C398.4A). The antibodies were purchased from SONY Biotechnology. Percentages of positive cells and median fluorescence intensity (MFI) values were determined according to autofluorescence and isotype‐matched antibody staining. The common gating strategy used for the immunophenotyping analyses is shown in Figure S1. The samples were run on a FACSAria II cell sorter (Becton Dickinson Biosciences) and analyzed by FlowJo software (Becton Dickinson Biosciences).

### 2.3 Cytokine secretion assay

The lymph node cells or PBMCs were stimulated with PMA (20 ng/mL) and ionomycin (0.5 μg/mL) (Sigma‐Aldrich) in MACS buffer for 4 h at 37°C. Then, the cells were washed with ice‐cold MACS buffer and labeled with IFN‐γ and TNF‐α catch reagents (Cytokine secretion assay kit, Miltenyi) for 10 min on ice. Following resuspension and incubation (50 min.) in warm culture media, the cells were washed with ice‐cold MACS buffer and further incubated with FITC‐conjugated IFN‐γ and APC‐conjugated TNF‐α detection antibodies (Cytokine secretion assay kit) (Miltenyi) for 10 min on ice. The cells were washed with ice‐cold MACS buffer, and CD8^+^ cells were gated and analyzed on a BD FACSCanto II flow cytometer (Becton Dickinson Biosciences, USA).

### 2.4 Statistical analysis

All statistical analyses were conducted with GraphPad Prism 8 (GraphPad Software, USA). The data obtained were presented as median and standard error. Normal distribution and homogeneity of variance were analyzed prior to Student's *t*‐test or one‐way ANOVA analyses where appropriate. The results were considered as statistically significant when the *p* value was <0.05.

## 3 RESULTS

### 3.1 Tfh‐like and T_EX_‐associated immunophenotype of CD8^+^ T cells in the regional lymph nodes

**FIGURE 1:**
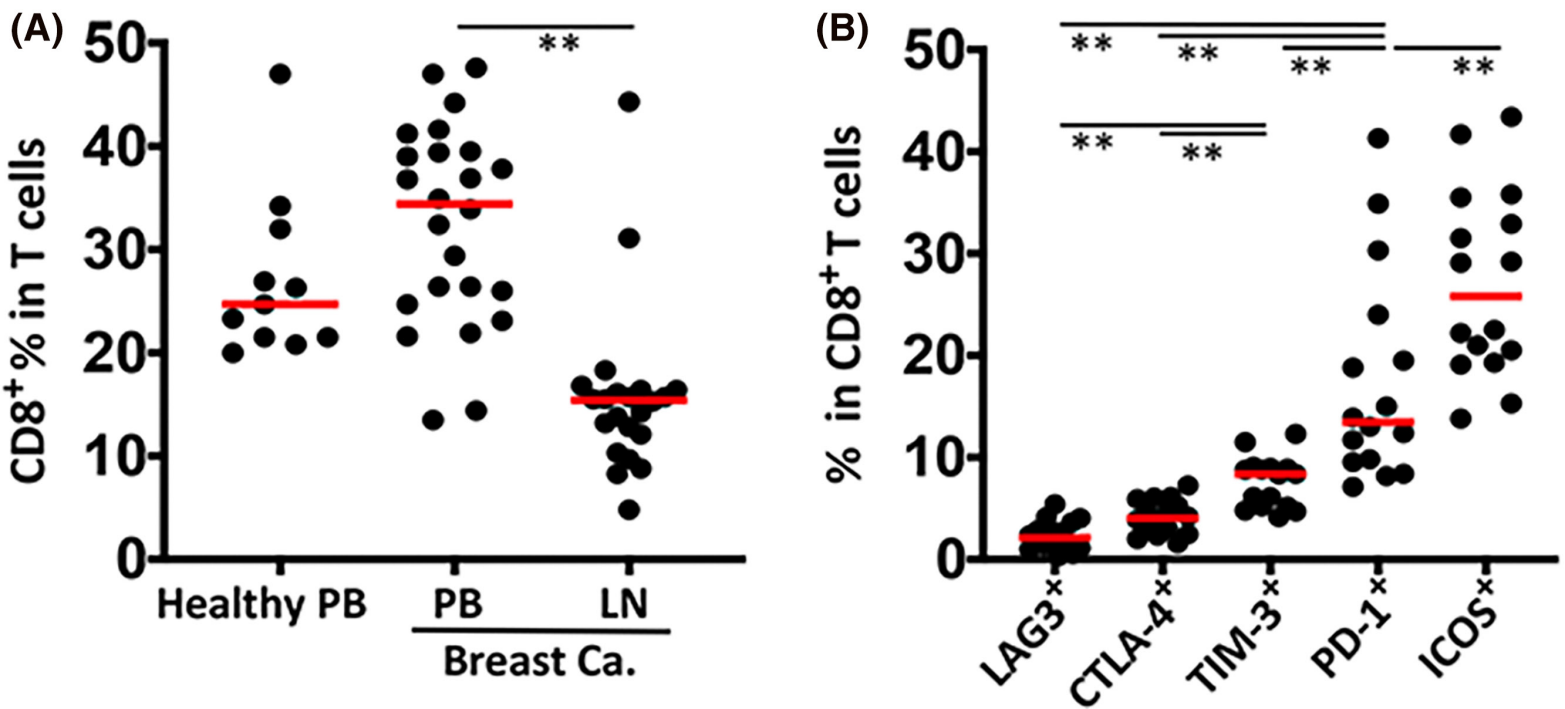
Expression of immune checkpoint receptors on CD8^+^ T cells in lymph nodes from breast cancer patients. (A) Percentage distribution of CD8^+^ T cells in peripheral blood (PB) samples from healthy individuals (*n* = 11) and breast cancer patients (*n* = 25), and in lymph node (LN) specimens from breast cancer patients (*n* = 22). The percentage of CD8^+^ T cells was determined by gating CD56^−^CD4^−^CD3^+^ parent population by flow cytometric immunophenotyping. The gating strategy is given in Figure S1. (B) Percentage of CD8^+^ T cells identified with the inhibitor receptors LAG3, CTLA‐4, TIM‐3, PD‐1, and the costimulatory receptor ICOS. The median value is designated with a red bar. Statistical difference was calculated with one‐way ANOVA, (**p ≤ 0.01). [Correction added on December 14, 2023 after first online publication. The upside down image of figure 1 has been set right in this version.]

In accordance with the literature, the percentage of circulating cytotoxic T cells was significantly higher (CD8^+^CD3^+^CD4^−^CD56^−^; healthy individuals, 27.11% ± 8.03%; breast cancer patients, 32.48% ± 9.65%) than the percentage of cytotoxic T cells in the lymph nodes (15.68% ± 8.12%) (Figure 1A). The expression of LAG3, CTLA‐4, TIM‐3, and PD‐1 inhibitory receptors and ICOS costimulatory receptor was assessed in the lymph node CD8^+^ T cells. CTLA‐4 and LAG3 were expressed only on a very small population of cytotoxic T cells (4.0%3 ± 1.66% and 2.11% ± 1.43%, respectively), whereas TIM‐3 (8.35% ± 2.47%), PD‐1 (13.45% ± 10.26%), and ICOS (27.80% ± 9.14%) expression were more frequently detected (Figure 1B).

**FIGURE 2:**
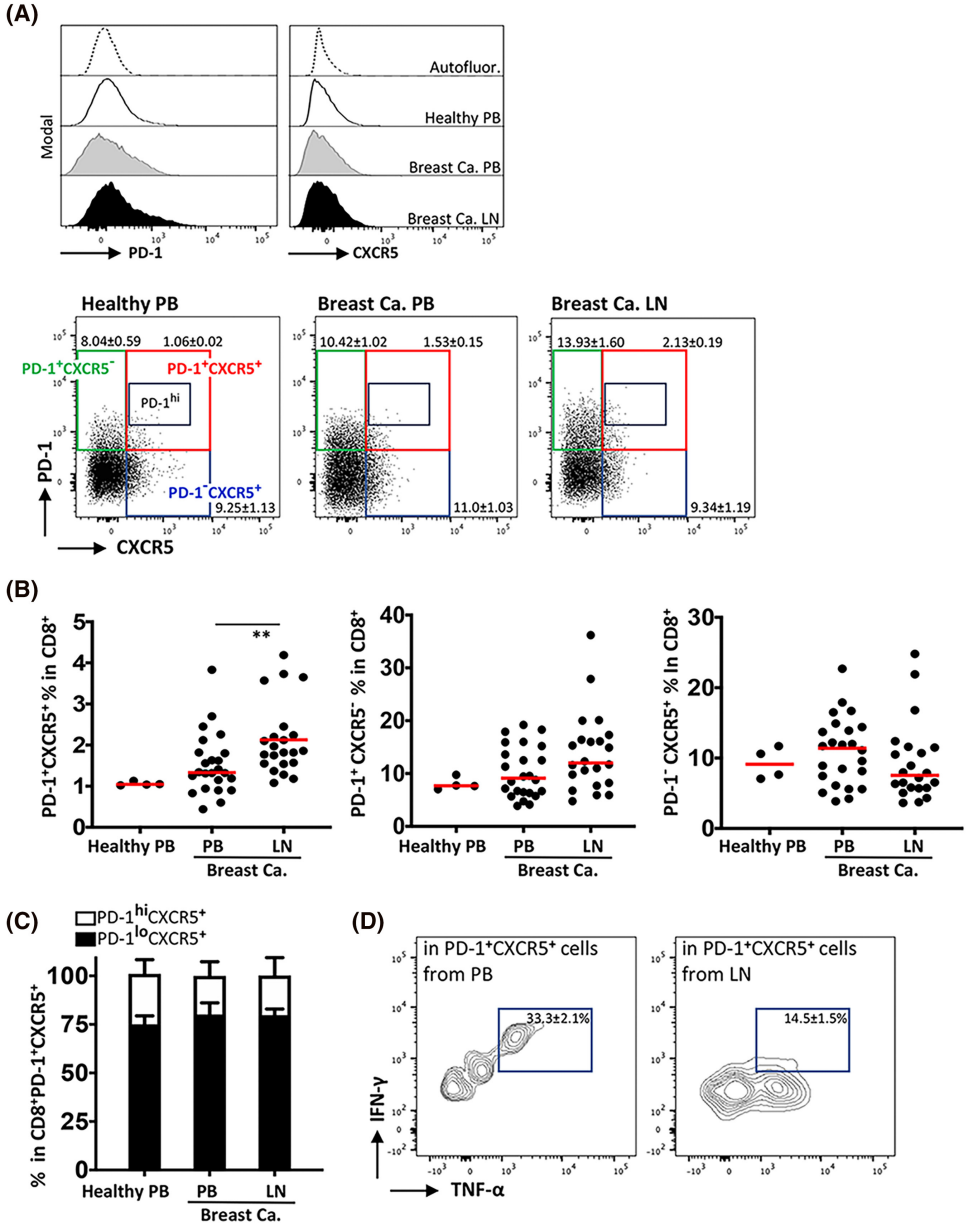
Determination of CD8^+^ T‐cell subpopulations according to PD‐1 and CXCR5 expression. (A) PD‐1 and CXCR5 expression was determined by flow cytometry on CD8+ T cells obtained from peripheral blood (PB) and lymph node (LN) samples from breast cancer patients (*n* = 19), and PB from healthy volunteers (*n* = 11). Representative offset histograms are given for each marker separately (upper panels) and flow cytometric dot plots displaying the co‐expression pattern of PD‐1 and CXCR5 on cytotoxic T cells (lowers panels). (B) Percent distribution of PD‐1^+^CXCR5^+^, PD‐1^+^CXCR5^−^, PD‐1^−^CXCR5^+^ subpopulations in CD8^+^ T cells. (C) Proportional percentage of PD‐1^+^CXCR5^+^ cytotoxic T cells with high‐level (PD‐1^hi^) and low‐level (PD‐1^lo^) expression of PD‐1 receptor. (D) IFN‐γ and TNF‐α secretion capacities of CD8^+^ T cells from PB and LN samples of breast cancer patients was assessed by a flow cytometric cytokine secretion assay. The median value is designated with a red bar. Statistical difference was calculated with one‐way ANOVA, (**p ≤ 0.01).

Since PD‐1, TIM‐3, and ICOS have been associated with Tfh‐like and T_EX_ cells, CD8^+^ T cells were distributed according to PD‐1 and CXCR5 expression. The PD‐1^+^CXCR5^−^ and PD‐1^−^CXCR5^+^ populations were notable in all compartments studied (Figure 2A). Albeit being a minor subpopulation, the percentage of PD‐1^+^CXCR5^+^ cytotoxic T cells (1.84% ± 0.88%) in the lymph nodes was significantly higher that of in the circulation of patients and healthy individuals (1.33% ± 0.75% and 1.05% ± 0.05%, respectively) (Figure 2A,B). Amongst the PD‐1^+^CXCR5^+^ cytotoxic T cells, approximately 25% of the cells were expressing high levels of PD‐1 (PD‐1^hi^), nevertheless no significant difference was found when the compartments where the cells obtained from (i.e., circulation and lymph nodes) or the data from healthy individuals and breast cancer patients were compared (Figure 2C). Moreover, compared to those obtained from the circulation of the cancer patients, the lymph node‐derived PD‐1^+^CXCR5^+^ cytotoxic T cells displayed considerable capacities to secrete TNF‐α and especially IFN‐γ (Figure 2D).

**FIGURE 3:**
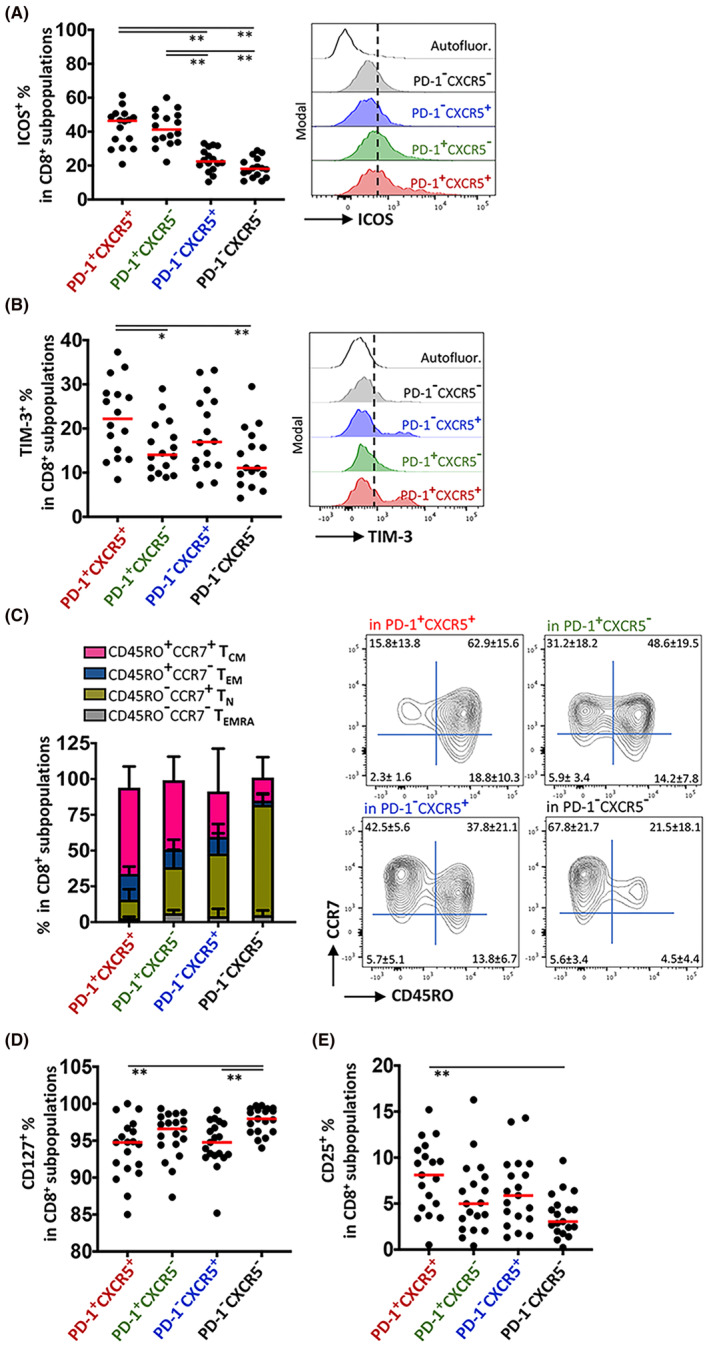
Assessment of Tfh‐like and memory‐like features of lymph node cytotoxic T‐cell subpopulations distributed according to PD‐1 and CXCR5 expression. (A) ICOS and (B) TIM‐3 expression on PD‐1^+^CXCR5^+^, PD‐1^+^CXCR5^−^, PD‐1^−^CXCR5^+^ and PD‐1^−^CXCR5^+^ subpopulations was determined by flow cytometry. Representative offset histograms are shown on the right‐hand side. (C) Central memory (T_CM_), effector memory (T_EM_), CD45RA^+^ effector memory (T_EMRA_) and naïve (T_N_) cells proportional distribution in cytotoxic T‐cell subpopulations distributed according to PD‐1 and CXCR5 expression. Representative flow cytometry dot plots are shown on the right‐hand side. The percentage of (D) CD127^+^ and (E) CD25^+^ cells in the subpopulations was determined by flow cytometry. The median value is designated with a red bar. Statistical difference was calculated with one‐way ANOVA, (*p ≤ 0.05, **p ≤ 0.01).

In the lymph nodes, ICOS was more frequently detected on the PD‐1^+^ cytotoxic T cells (range, 20.80%–61.40%), than on the PD‐1^−^ cytotoxic T cells (range, 10.40%–33.10%) which was independent of the CXCR5 positivity (Figure 3A). On the other hand, the percentages of TIM‐3^+^ cells (22.20% ± 8.58%) were significantly high in the PD‐1^+^CXCR5^+^ fraction (Figure 3B and Figure S2). More than ~62% of the PD‐1^+^CXCR5^+^ cytotoxic T cells displayed central memory T (T_CM_) cell markers CCR7 and CD45RO. The percentage of T cells with T_CM_ markers showed a decreasing trend; they were the most frequently found in PD‐1^+^CXCR5^+^, then in PD‐1^+^CXCR5^−^, followed by PD‐1^−^CXCR5^+^ and PD‐1^−^CXCR5^−^ fractions. Alternatively, the percentage of naïve T cells (T_N_, CD45RO^−^CCR7^+^) had a trend opposite to that of T_CM_ amongst the subpopulations of CD8^+^ T cells distributed according to PD‐1 and CXCR5 (Figure 3C). Next, we compared the TNF‐α and IFN‐γ secretion capacity amongst the total CD8^+^ T_CM_ cells, the total PD1^+^CXCR5^+^ CD8^+^ T cells, and the T_CM_ fraction of the PD1^+^CXCR5^+^ CD8^+^ T cells. In accordance with the finding that a large portion of PD1^+^CXCR5^+^ CD8^+^ T cells were displaying T_CM_ phenotype (Figure 3C), the T_CM_ cells with PD1^+^CXCR5^+^ exhaustion phenotype harbored decreased percentage of cells with IFN‐γ and/or TNF‐α (Table 2). The cytokine secretion by the total CD8^+^ T_CM_ population was significantly higher than the CD8^+^ T cells bearing the exhaustion markers (Table 2).

**TABLE 2:** Percentage of the cells with TNF‐α and/or IFN‐γ secretion capacity in CD8^+^ TCM cells, PD1^+^CXCR5^+^ CD8^+^ T cells and PD1^+^CXCR5^+^ CD8^+^ T_CM_ cells.

	In total CD8^+^ T_CM_ cells	In PD1^+^CXCR5^+^ CD8^+^ T cells	In PD1^+^CXCR5^+^ CD8^+^ T_CM_ cells
IFN‐γ^+^ (%)	27.8 ± 5.6	16.4 ± 1.1^a^	25.8 ± 1.5
TNF‐α^+^ (%)	71.5 ± 13.9	39.1 ± 5.8	27.5 ± 6.5
IFN‐γ^+^TNF‐α^+^ (%)	19.7 ± 1.9	7.7 ± 1.7^a^	7.9 ± 0.8^a^

*Note*: T_CM_ means central memory T cells; *n* ≤ 3.

^a^ *p* ≤ 0.05.

Albeit being expressed on more than 85% of the cells, the percentage of CXCR5^+^ cells expressing the IL‐7 receptor alpha (CD127) was significantly decreased when compared to that of PD‐1^−^CXCR5^−^ T cells which harbored a large percentage of naïve T cells (Figure 3D). On the other hand, IL‐2 receptor alpha (CD25) was significantly but heterogeneously upregulated on a small percentage of PD‐1^+^CXCR5^+^ cells (range, 0.51%–15.20%). The cytotoxic T‐cells subpopulations other than PD‐1^+^CXCR5^+^ cells displayed less numbers of CD25^+^ cells (Figure 3E).

Collectively, in the circulation and especially in the regional lymph nodes of breast cancer patients, a subpopulation of CXCR5^+^PD‐1^+^ cytotoxic T cells was frequently identified with T_CM_ phenotype, expression of TIM‐3 and ICOS receptors, and had reduced capacity to secrete IFN‐γ.

### 3.2 The change in PD‐1^+^ cytotoxic T‐cell subpopulations associated with preoperative neoadjuvant chemotherapy in breast cancer patients

The percentages of CD8^+^ T‐cell populations distributed according to CXCR5 and PD‐1 expression were analyzed together with the patient records such as clinical stage, histopathological grade, HER2 status, neoadjuvant therapy prior to the surgery, and therapy response upon completion of all medical interventions. In general, the clinical data of the patients showed limited association with the CD8^+^ T‐cell populations determined (Table 3). No significant difference was obtained when the lymph node metastasis scores (N0, N1(mi), or N1≤) were considered amongst the patients. Only, the percentage of PD‐1^+^CXCR5^−^ cells in the lymph node tends to significantly decrease when compared to the patients underwent neoadjuvant therapy (15.3% ± 7.48% in the treatment‐naïve patients and 5.92% ± 3.11% in the neoadjuvant group) (Table 3). Moreover, the percentage of PD‐1^−^CXCR5^+^ cells tended to be higher in the lymph node samples of patients in the stage I–II patients (7.96% ± 5.65%) than those were in the stage III (4.32% ± 1.11%) (Table 3). Nevertheless, the limited number of patients enrolled to the study should be considered as a drawback for reaching statistically significant results.

**TABLE 3:** Distribution of CD8^+^ T‐cell CXCR5 and PD‐1 subpopulations according to patients' clinical properties (average ± SEM).

	*n*	PD‐1^+^CXCR5^+^ (%)	PD‐1^+^CXCR5^−^ (%)	PD‐1^−^CXCR5^+^ (%)
Therapy				
LN naïve	17	1.97 ± 0.93	15.30 ± 7.48^a^	6.54 ± 4.48
LN neoadjuvant	5	1.77 ± 0.45	5.92 ± 3.11^a^	10.70 ± 7.53
PB naïve	19	1.32 ± 0.77	9.44 ± 4.51	9.58 ± 4.88
PB neoadjuvant	5	1.63 ± 0.72	6.31 ± 6.24	13.90 ± 4.12
Therapy response follow‐up				
LN stable	15	2.21 ± 0.92	14.71 ± 8.56	7.87 ± 3.31
LN progressed	7	1.96 ± 0.80	12.26 ± 4.58	12.49 ± 8.16
PB stable	18	1.72 ± 0.79	9.88 ± 4.85	12.07 ± 4.71
PB progressed	6	1.14 ± 0.46	11.00 ± 5.04	8.68 ± 4.60
Clinical stage				
LN (0‐II)	19	1.97 ± 0.93	12.30 ± 8.03	7.96 ± 5.65^a^
LN (III)	3	1.75 ± 0.13	11.70 ± 2.91	4.32 ± 1.11^a^
PB (0‐II)	21	1.34 ± 0.78	8.79 ± 4.95	11.70 ± 4.93
PB (III)	3	1.32 ± 0.40	9.54 ± 4.78	5.06 ± 4.39
Histopathological grade				
LN (I–II)	16	1.84 ± 0.93	11.10 ± 8.77	7.54 ± 6.22
LN (III)	6	1.90 ± 0.80	13.80 ± 2.38	9.02 ± 3.71
PB (I–II)	16	1.59 ± 0.56	8.96 ± 4.83	11.95 ± 4.86
PB (III)	8	1.08 ± 1.05	10.65 ± 5.04	7.59 ± 4.24
HER2 status				
LN positive	5	2.12 ± 1.07	12.77 ± 4.28	14.90 ± 6.98
LN negative	17	1.82 ± 0.81	14.27 ± 8.29	11.70 ± 5.31
PB positive	6	1.33 ± 0.55	10.98 ± 5.21	10.49 ± 5.31
PB negative	18	1.37 ± 0.81	10.01 ± 4.83	9.12 ± 4.85

Abbreviations: LN, lymph node; PB, peripheral blood.

^a^ *p* ≤ 0.05.

## 4 DISCUSSION AND CONCLUSION

The information on antitumor immune responses, which are commonly dysregulated in cancer patients, are generally obtained from the circulating leukocytes.^29^ Nevertheless, the lymph nodes in vicinity to the tumor tissue, wherein the antigen presentation and stimulation of T cells take place, represent a superior specimen for understanding the immune regulation on the lymphocytes.^28^ In this study, we focused on the immune checkpoint expression status and exhaustion‐related features of CD8^+^ cytotoxic T cells in the regional lymph nodes of breast cancer patients. Compared to the peripheral blood, the lymph nodes harbor a significant number of cytotoxic T cells with an exhausted phenotype. In the regional lymph nodes, the co‐expression of PD‐1, CXCR5, TIM‐3, and ICOS has marked a subpopulation that displayed a reduced capacity to produce IFN‐γ. The immunological status of cytotoxic T cells in the regional lymph nodes should be considered while assessing the immune competency. Therefore, identification of the subpopulation CXCR5^+^PD‐1^+^ cytotoxic T cells which frequently expressed TIM‐3, in the lymph nodes may be important while assessing suitability of cancer patients for immune checkpoint immunotherapies (ICI) targeting PD‐1 and TIM‐3 pathways. According to our data, it might be speculated that ICI can act on the regional lymph nodes containing a CXCR5^+^PD‐1^+^ population and might foster the antitumor immunity through rejuvenation of exhausted T cells.

In the secondary lymphoid organs, CXCR5 and ICOS mark a specialized subset of CD4^+^ T cells, acknowledged as the T follicular helper cells (Tfh), which play a key role in protective immunity by contributing to the formation of germinal centers and assisting B cells to produce antibodies.^19^ ICOS is expressed on activated T cells, and its ligand B7‐H2 (ICOSLG) is found on B cells.^30^ Even though ICOS has been functionally designated as a costimulatory molecule providing signals for T‐cell activation and proliferation, ICOS^−/−^ mice are susceptible to autoimmunity.^31^ This may indicate a possible regulatory role for ICOS on T‐cell immunity. A subset of Tfh cells has been recognized with the expression of PD‐1 and TIM‐3 inhibitory receptors.^20^ On the other hand, these markers have been associated with the hyporesponsiveness in CD8^+^ cytotoxic cells. In CD8^+^ T cells, PD‐1 and CXCR5 are emblematic surface molecules for progenitor‐exhausted T cells, whereas PD‐1 and TIM‐3 indicate a terminal exhaustion.^18^ In addition, the progenitor‐exhausted T cells have been recognized as a memory‐like subset. This notion was supported by our findings in the lymph nodes since PD1^+^CXCR5^+^ cytotoxic T cells highly displayed CD45RO and CCR7 T_CM_ associated molecules. Nevertheless, T_CM_ markers were also frequently detected on the PD1^+^CXCR5^−^ subpopulation. As a drawback of our study, the expression of exhaustion‐related key transcription factors TCF‐1, Tox, and Eomes were not tested^32^, ^33^, ^34^; here, the immunophenotypic features of T cells, which are related with the exhaustion, were prioritized in addition to the functional cytokine secretion capacities. Moreover, we did not compare circulating and lymph node PD1^+^CXCR5^+^ cytotoxic T‐cell subpopulations in terms of additional markers related to exhaustion and memory features.

Several studies regarded the CXCR5^+^CD8^+^ T cells to be functionally analogous to Tfh, found in the lymphoid structures of colorectal cancer patients.^25^, ^26^ CD8^+^ Tfh cells were observed with immunohistochemistry or immunofluorescence techniques in the tumor microenvironment and the tumor‐draining lymph nodes.^35^ Xing et al. reported the expression of Bcl‐6, which is the signature transcription factor for Tfh, in CXCR5^+^CD8^+^ T cells.^26^ Nevertheless, there is no direct evidence demonstrating the functional contribution of CD8^+^ T cells on B cell responses in the germinal centers. Alternatively, by using a multiparametric immunophenotyping approach, we were able to associate CXCR5^+^CD8^+^ T cells with an exhausted state in breast cancer. The exhausted cytotoxic T cells residing in the tumor‐draining regional lymph nodes may possess pivotal importance for immune checkpoint inhibitor (ICI) therapies. The blockade of PD‐1, which is the most acknowledged target for ICI, had limited success in breast cancer.^7^ In accordance with our results and findings from another study,^4^ TIM‐3 may serve as an alternative checkpoint on the cytotoxic T cells hindering the antitumor immune responses.

In breast cancer, a significant relationship between CXCL13 levels, and unfavorable clinical features, such as the status of lymph node involvement, has been reported.^36^ Especially, CXCL13/CXCR5 axis was associated with axillary lymph node metastases.^37^ CXCR5 serves as a receptor for the chemokine CXCL13, which is highly expressed in the lymphoid follicles and provides B and T cell compartmentalization for germinal center reactions.^38^ It might be speculated that a circulating CXCR5^+^ T cell would eventually arrive at the lymphoid tissues and reside nearby the B cell zone. This migratory property of CXCR5^+^ T cell might increase their likelihood to become more frequently exposed to cognate antigens and the immune modulatory factors derived from the tumor microenvironment. Therefore, in the regional or tumor‐draining lymph nodes, the cytotoxic T cells may be more prone to immune exhaustion.

The peripheral blood serves as the major source of leukocytes cells for immunological analyses in humans.^29^ Alternatively, our data demonstrated that the cytotoxic T cells from the regional lymph nodes in cancer were under a larger immune modulatory pressure when compared to the cells obtained from the circulation. From this point of view, the regional lymph nodes may serve as a target organ for designing immunotherapeutic interventions. To our knowledge, this is the first study that considers associating the clinical features of breast cancer patients with the immune exhaustion and immune checkpoint molecule expression status in the lymph nodes. Since the immunological parameters analyzed were not largely associated with the clinical data, we concluded that the T‐cell exhaustion may exist in the lymph nodes, independent of clinical or histopathological grade, and hormone receptor status. Intriguingly, the neoadjuvant (preoperative) chemotherapy significantly tended to decrease the level of PD‐1^+^ cytotoxic T cells in the regional lymph nodes. The neoadjuvant chemotherapy has been used as the standard of care for the breast cancer patients with massive primary tumors or complicated lymph node metastases.^39^ The patients benefit from successful tumor downstaging, local control of disease and demonstrated increased response to chemotherapy.^40^ Therefore, it may be speculated that the reduction in the tumor burden following neoadjuvant chemotherapy might have also led to decrease in the numbers of exhausted T cells in the regional lymph nodes. Dynamic changes in the immune infiltration status of primary tumors, such as increase in PD‐1^+^CD8^+^ T cells, have been previously reported after neoadjuvant therapy in breast cancer.^41^ Nevertheless, further studies with larger study cohorts are needed to better understand the relationship between tumor burden, therapy response, and immune exhaustion.

The T cell‐mediated antitumor immune responses are initiated and expanded in the regional tumor‐draining secondary lymphoid organs.^8^ As a primary site for antigen presentation, a better description of the immune status in the mammary lymph nodes of breast cancer patients is pivotal for understanding the intensity of immune modulation in the tumor microenvironment.^28^ Therefore, our findings on lymph node CD8^+^ T cells can contribute to the understanding of immune modulation in breast cancer. Notably, the presence of T‐cells bearing targets for ICI in the tumor‐draining lymph nodes must be considered while assessing the immune competency of the patient and during decision‐making for immunotherapeutic interventions.

## AUTHOR CONTRIBUTIONS

**Izel Yilmaz:** Conceptualization (equal); methodology (equal); writing – original draft (equal); writing – review and editing (equal). **Ece Tavukcuoglu:** Investigation (equal); methodology (equal). **Utku Horzum:** Investigation (equal); methodology (equal). **Kerim Bora Yilmaz:** Investigation (equal); methodology (equal). **Melih Akinci:** Investigation (equal); methodology (equal). **Mehmet Ali Gulcelik:** Investigation (equal); methodology (equal). **Haluk Barbaros Oral:** Conceptualization (equal); project administration (equal); writing – review and editing (equal). **Gunes Esendagli:** Conceptualization (equal); project administration (equal); supervision (equal); writing – original draft (equal); writing – review and editing (equal).

## FUNDING INFORMATION

The authors affirm that no funds, grants, or other support were received during preparation of this manuscript.

## CONFLICT OF INTEREST STATEMENT

None declared.

## ETHICAL APPROVAL

This study was performed in line with the principles of the Declaration of Helsinki. Approval was granted by the Ethics Committee of University of Health Sciences Gulhane Training and Research Hospital (Date 11.05.2020/No 46418926). Informed consent was obtained from all participants.

## Supporting information


Figures S1–S2.

## Data Availability

For original data please contact gunese@hacettepe.edu.tr.
