# Supplementary material for: Immune checkpoint status and exhaustion‐related phenotypes of CD8+ T cells from the tumor‐draining regional lymph nodes in breast cancer
Source: Cancer Med. 2023 Dec 8;12(24):22196–205. doi: 10.1002/cam4.6802 (PMC10757146; doi:10.1002/cam4.6802)
Supplement: Supplementary file 1 — Figures S1–S2. [file CAM4-12-22196-s001.pdf]

Supplementary figures

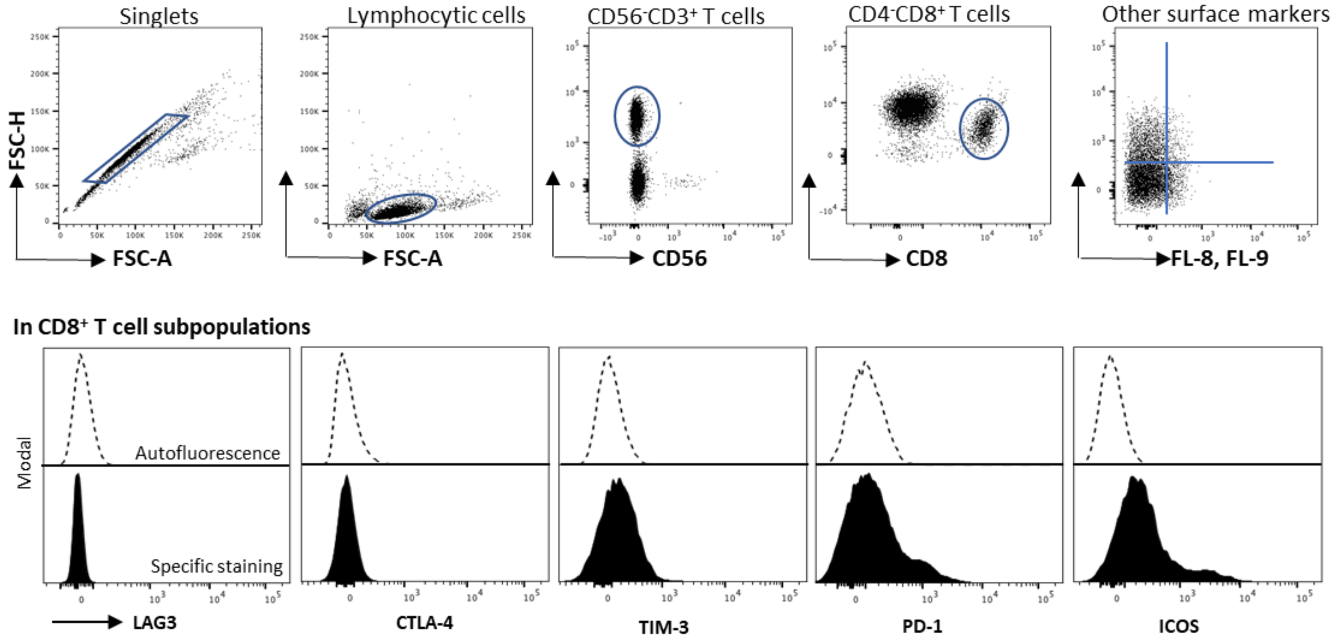

Suppl. Fig. 1. The gating strategy used for immunophenotyping by flow cytometry (upper panel) is shown together with representative histograms for immune checkpoint receptor expression in cytotoxic T cells (lower panel).

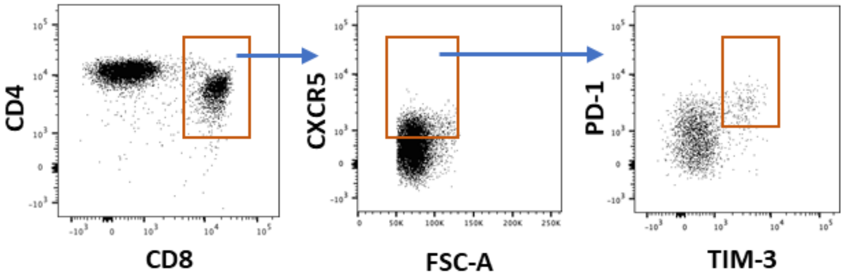

Suppl. Fig. 2. The gating strategy used for determining the TIM-3 and PD-1 expression on CXCR5-positive cytotoxic $CD8^+$ T cells from the lymph nodes. Representative dot plots from an immunophenotyping analysis by flow cytometry are demonstrated. The orange square on the left-hand-side plot shows a subpopulation of cells co-expressing TIM-3 and PD-1.
